# Food restriction reduces neurogenesis in the avian hippocampal formation

**DOI:** 10.1371/journal.pone.0189158

**Published:** 2017-12-06

**Authors:** Barbara-Anne Robertson, Lucy Rathbone, Giselda Cirillo, Richard B. D’Eath, Melissa Bateson, Timothy Boswell, Peter W. Wilson, Ian C. Dunn, Tom V. Smulders

**Affiliations:** 1 Institute of Neuroscience, Newcastle University, Newcastle upon Tyne, United Kingdom; 2 Centre for Behaviour and Evolution, Newcastle University, Newcastle upon Tyne, United Kingdom; 3 School of Psychology, Newcastle University, Newcastle upon Tyne, United Kingdom; 4 Animal Behaviour and Welfare Group, Scotland’s Rural College, Easter Bush, United Kingdom; 5 School of Natural and Environmental Sciences, Newcastle University, Newcastle upon Tyne, United Kingdom; 6 Roslin Institute, University of Edinburgh, Easter Bush, United Kingdom; University of Modena and Reggio Emilia, ITALY

## Abstract

The mammalian hippocampus is particularly vulnerable to chronic stress. Adult neurogenesis in the dentate gyrus is suppressed by chronic stress and by administration of glucocorticoid hormones. Post-natal and adult neurogenesis are present in the avian hippocampal formation as well, but much less is known about its sensitivity to chronic stressors. In this study, we investigate this question in a commercial bird model: the broiler breeder chicken. Commercial broiler breeders are food restricted during development to manipulate their growth curve and to avoid negative health outcomes, including obesity and poor reproductive performance. Beyond knowing that these chickens are healthier than fully-fed birds and that they have a high motivation to eat, little is known about how food restriction impacts the animals' physiology. Chickens were kept on a commercial food-restricted diet during the first 12 weeks of life, or released from this restriction by feeding them *ad libitum* from weeks 7–12 of life. To test the hypothesis that chronic food restriction decreases the production of new neurons (neurogenesis) in the hippocampal formation, the cell proliferation marker bromodeoxyuridine was injected one week prior to tissue collection. Corticosterone levels in blood plasma were elevated during food restriction, even though molecular markers of hypothalamic-pituitary-adrenal axis activation did not differ between the treatments. The density of new hippocampal neurons was significantly reduced in the food-restricted condition, as compared to chickens fed *ad libitum*, similar to findings in rats at a similar developmental stage. Food restriction did not affect hippocampal volume or the total number of neurons. These findings indicate that in birds, like in mammals, reduction in hippocampal neurogenesis is associated with chronically elevated corticosterone levels, and therefore potentially with chronic stress in general. This finding is consistent with the hypothesis that the response to stressors in the avian hippocampal formation is homologous to that of the mammalian hippocampus.

## Introduction

Once thought to be restricted to early neural development, neurogenesis is now widely accepted to occur in adult vertebrate brains, including in birds and mammals. In adult mammals, new neurons are added to the dentate gyrus region of the hippocampus, and the olfactory bulb [1–4]. Neurogenesis in birds occurs all over the telencephalon [5], and is widely accepted to be an ongoing process across the life span [2]. Neurogenesis is a multistage process in which new neurons are born (proliferation), move to specific brain regions (migration), and then are incorporated into existing brain circuits through dendritic growth and forming synapses (differentiation) [2]. The proliferation of new cells which will develop into neurons occurs in the subventricular zone (neurons destined for the olfactory bulb) and the subgranular zone (in the dentate gyrus) in mammals [6]. In birds, proliferation of neuronal progenitors happens in the ventricular zone [7, 8] in particular ‘hot spots’ in dorsal and ventral portions of the lateral ventricle wall [9].

Stressors are challenges to an organism that create internal adverse effects. Stress is defined as an actual or perceived threat to homeostasis; homeostasis is then re-established through adaptive behavioural and physiological responses [10]. Stress can have profound effects on hippocampal morphology, connectivity, and function [11, 12]. If stress is chronic, it can lead to hippocampal atrophy. This is thought to result from long-term glucocorticoid dysregulation [13]. Hippocampal volumetric reduction between 8%–19% is reliably reported in studies of humans diagnosed with Major Depressive Disorder [14–17], and in rodents exposed to chronic stress [15, 18].

Neurogenesis in the mammalian hippocampus is sensitive to the effects of stressors [11, 19, 20]. The stress-induced suppression of hippocampal neurogenesis is especially pronounced in the ventral (also called temporal), and less in the dorsal (also called septal) hippocampus [21]. This is consistent with other evidence suggesting that the dorsal and ventral hippocampus serve separate functions, with the ventral hippocampus being especially involved in the regulation of the stress response [22]. In the mammalian hippocampus, chronic stress negatively impacts neurogenesis [23, 24] via stimulation of glucocorticoid receptors [11, 12]. This effect is mediated, at least in part, by altering the function of the hypothalamic-pituitary-adrenocortical (HPA) axis and the mechanisms which support it [12, 19, 25–28]. Glucocorticoid hyperfunction can reduce adult hippocampal neurogenesis [19, 28], but glucocorticoid hypofunction is also damaging: low doses of corticosterone (CORT) replacement post-adrenalectomy are required in rats to avoid apoptotic loss of dentate gyrus neurons [29]. Increases in CORT above baseline levels do not always suppress adult hippocampal neurogenesis, however. Under some conditions, they can actually increase neurogenesis, suggesting that CORT impacts the hippocampus in a complex and bidirectional manner [30]. Therefore, it would seem that CORT release is not damaging in itself, but rather it is the conditions which stimulate CORT release that seem to dictate whether the effects on the hippocampus will be restorative or damaging.

The avian hippocampal formation (HF) is homologous to the mammalian structure, as evidenced by developmental patterns [31] and connectivity [32–34], although its cyto-architecture is strikingly different [2, 35, 36]. Functionally, the avian HF is similar to its mammalian equivalent, being involved in spatial memory and navigation [37–40]. Like the mammalian hippocampus, the avian HF is also involved in the regulation of the HPA axis [41, 42]. It expresses both glucocorticoid (GR) and mineralocorticoid (MR) receptors [43–47] and, like in rats, increased CORT levels and/or stress lead to a down-regulation of MR receptors in the avian HF [43, 44, 48]. As in mammals, avian hippocampal morphology and neurogenesis are sensitive to environmental stressors. Captivity shrinks hippocampal volume in wild-caught songbirds [37, 49, 50], and a number of stressors have been shown to decrease adult hippocampal neurogenesis in birds [51–53]. At present, there is no accepted subdivision of the avian HF that is equivalent to the dorsal-ventral distinction in rodents. However, based on topological arguments, it has recently been suggested that the rostral avian HF may be equivalent to the dorsal hippocampus in rodents, and the avian caudal pole to the rodent ventral pole [42].

Caloric restriction is a potential stressor, and can affect hippocampal neurogenesis. Interestingly, the effect of caloric restriction on hippocampal neurogenesis seems to depend on the developmental stage of the animals and/or on the pattern of food availability. In adult rats and mice fed *ad libitum* every other day (and completely food deprived on the alternate days), caloric restriction leads to an increase [54–57], while in adolescent rats, fed 60% of *ad libitum* for 2 months, caloric restriction leads to a decrease in hippocampal neurogenesis [58]. To date, nothing is known about the effects of food restriction on hippocampal neurogenesis in birds.

Our model system is that of commercial broiler breeder hens (*Gallus gallus domesticus*). These birds are the parent stock of meat chickens produced for consumption. During rearing to adulthood, it is common industrial practice to restrict the amount of the birds’ food to 33%–40% of what they would eat if provided with *ad libitum* food [59, 60]. This restriction results in hunger, shown behaviourally by increased foraging [61] and stronger motivation to overcome adverse stimuli to obtain food [62], and physiologically by the upregulation of expression of orexigenic neuropeptide mRNAs in the hypothalamus [63]. However, food restriction also avoids many of the serious negative health consequences experienced by broiler chickens fed *ad libitum* including reduced fertility, double ovulation, lameness, heart failure, thermal dysregulation and increased mortality due to skeletal and metabolic disease [64]. This situation results in what has been called the Broiler Breeder Paradox [65, 66] and has been highlighted as a welfare dilemma [60, 61, 67]. Despite having their food severely restricted, broiler breeders gain weight, grow and are reproductively viable like non-broiler genotypes [68].

The purpose of this study was therefore to determine the effect of chronic food restriction on avian hippocampal neurogenesis in adolescent broiler breeder chickens, and to assess whether such an effect is stronger in the rostral or caudal pole of the HF. In addition to measuring hippocampal neurogenesis, we also looked for markers of activation of the HPA axis and its consequences.

## Materials and methods

### Animals

Twenty-four female broiler breeder chickens (Ross 308 line) were housed in groups of three, across eight replicated pens. They were maintained and fed according to the 2007 Aviagen Ross 308 management manual for broiler breeders (http://en.aviagen.com/ross-308/). Briefly, a starter (19% crude protein) and a grower diet (15% crude protein) with an energy density of 11.7 MJ/kg was fed from 0–4 and 4–12 weeks of age, respectively. In the standard commercial protocol, these diets are available *ad libitum* from 0–1 weeks and thereafter stepwise to 44 g/bird/day at 6 weeks of age and 58 g/bird/day by 12 weeks of age. Half the birds were kept on this regimen for the 12-week duration of the experiment (Food-Restricted or FR; n = 12). The other half were released from restriction when 6 weeks old and fed the same diet *ad libitum* (AL; n = 12) until 12 weeks old. To summarize, AL birds were food restricted from 1 week to 6 weeks old, and fed *ad libitum* from 6 weeks to 12 weeks old, and FR birds were food restricted from 1 week to 12 weeks old. Animals used in this study were the same subjects used by Dunn *et al*. [63]], Experiment One (see that study for protocols detailing exact feeding, housing and husbandry regimes). At 11 weeks of age, two bromodeoxyuridine (BrdU) injections were administered subcutaneously on two successive days: first at a dose of 100mg/kg (solution of 50mg/ml), and then 87.9mg/kg the next day.

### Tissue collection

The experiment was performed under a UK Home Office Project Licence PPL 60/3964. Blood samples were taken from the brachial vein in the afternoon of the three last days of the birds’ lives. Birds were taken out of the pen and into another room for blood sampling, after which they were returned to the pen (except on the last day). Birds from the two treatments were alternated. The restricted birds were fed in the morning, so that they were sampled after they had fed, and long before they expected to be fed again. We report hormone titres from the last sample, when birds had most habituated to the blood sampling procedure. Right after the last sample, the birds were humanely killed (intravenous injection of 1–2 mL of Sodium Pentobarbital (200 mg/mL) into a brachial wing vein) as specified in Schedule 1 of the UK Animals (Scientific Procedures) Act 1986. The animals were weighed and their tarsometatarsal length was measured. The adrenal glands, spleen, pituitary gland, basal hypothalamus and right HF were dissected immediately after death, weighed, frozen in liquid nitrogen, and stored at -80°C until RNA was extracted. The left hemisphere was fixed in a solution of 5% acrolein and phosphate buffered saline (PBS) for one hour, then into a fresh solution of 5% acrolein and PBS for a second hour, followed by a 30% sucrose solution until the tissue was saturated and sank. Hemispheres were then embedded in OCT Tissue-Tek®, quickly frozen in a mix of 95% ethanol and dry ice before being stored at -80°C until the tissue was sectioned in preparation for immunohistochemistry.

### Immunohistochemistry

From the left hemisphere, 50 *μ*m coronal sections were cut on a Microm HM560 cryostat into 0.1 M Phosphate Buffered Saline, 7.4 pH (PBS) before being stored in a cryoprotectant solution (20% 0.3M PBS, 30% Ethylene Glycol, 30% Glycerol, and 20% deionised water) at -20°C. When ready for processing, the tissue was stained first for BrdU as a marker of newly-generated cells and was then counterstained with an antibody against the Elav-like neuronal-specific protein Hu [69].

#### BrdU Labelling

Every fourth section (200 *μ*m apart) was removed from cryoprotectant into 0.1 M PBS and then washed 3 x 5 min. Free-floating sections were incubated for 15 min in 0.001% NaBH_4_, followed by 3 x 2min washes in 0.1M PBS. To reduce endogenous peroxidase activity, sections were incubated in 0.3% H_2_O_2_ for 10 minutes before again being washed 3 x 2min in 0.1M PBS. BrdU substitutes for thymidine during S-phase of DNA replication. To access this in the DNA, denaturation is required before immunolabelling. Sections were placed in 2N HCl for 30 minutes in a water bath held at 37°C. This was followed by 3 x 2 minute 0.1M PBS washes. Sections were incubated for 60 minutes in blocking solution (10% Normal Goat Serum (NGS) in PBS + 0.3% TritonX (PBSx)) before being washed (3 x 2min 0.1M PBS). Sections were then incubated overnight at 4°C in 1:3000 anti-BrdU primary antibody solution (AbD Serotec Cat# MCA2060T Lot# RRID:AB_10015293; rat anti-BrdU OBT0030, Batch#0109 AbD Serotec, and 0.25% NGS in 0.3% PBSx). The next morning, the tissue was washed thrice in 0.1M PBS before being incubated for 120 min in 1:1000 biotinylated secondary antibody (anti-rat IgG (H+L) made in goat (BA-9400 Vector Labs UK) in 0.1%PBSx), then washed thrice in 0.1M PBS followed by 60 min in 1:200 HRP-streptavidin conjugate (Vector Labs UK SA-5000) in 0.1%PBSx. After 3 x 2 minute 0.1M PBS washes, the tissue was stained using the chromogen Slate Grey (Vector Labs UK SK-4700) for 3 minutes before the reaction was stopped with tap water, following the recommended product instructions. The tissue was then washed in 0.1M PBS (3 x 2 min).

#### Hu labelling

Washed sections were left to incubate overnight at 4°C in anti-Hu primary antibody solution (1:3000; anti-HuC/HuD mouse IgG_2b_, Invitrogen A21271; with 0.37% NGS in 0.3% PBSx). On the following morning, the tissue was washed thrice in 0.1M PBS before being incubated at room temperature for 120 min in 1:1000 biotinylated secondary antibody (anti-mouse IgG(H+L) made in goat; Vector Labs UK BA-9200 in 0.1% PBSx), then washed thrice in 0.1M PBS followed by 60 min in 1:200 HRP-streptavidin conjugate (Vector Labs UK SA-5000) in 0.1% PBSx. After 3 x 2 minute 0.1M PBS washes, the tissue was stained using the chromogen Nova Red (Vector Labs UK SK-4800) for 3.5 minutes before the reaction was stopped with tap water for 5 min. The tissue was washed in 0.1M PBS (3 x 2 min), and was stored in the final wash until mounted onto slides.

Sections were floated in deionized water and were individually mounted onto gelatine-covered slides using a paint brush. Slides were left to air dry overnight before being cleared for 2 minutes in Histoclear®, removed, and immediately covered in Histomount® and cover glass. Slides were left until the mounting medium set, were cleaned of excess medium and were then ready for microscopic investigation.

### Microscopy analysis

**Fig 1:**
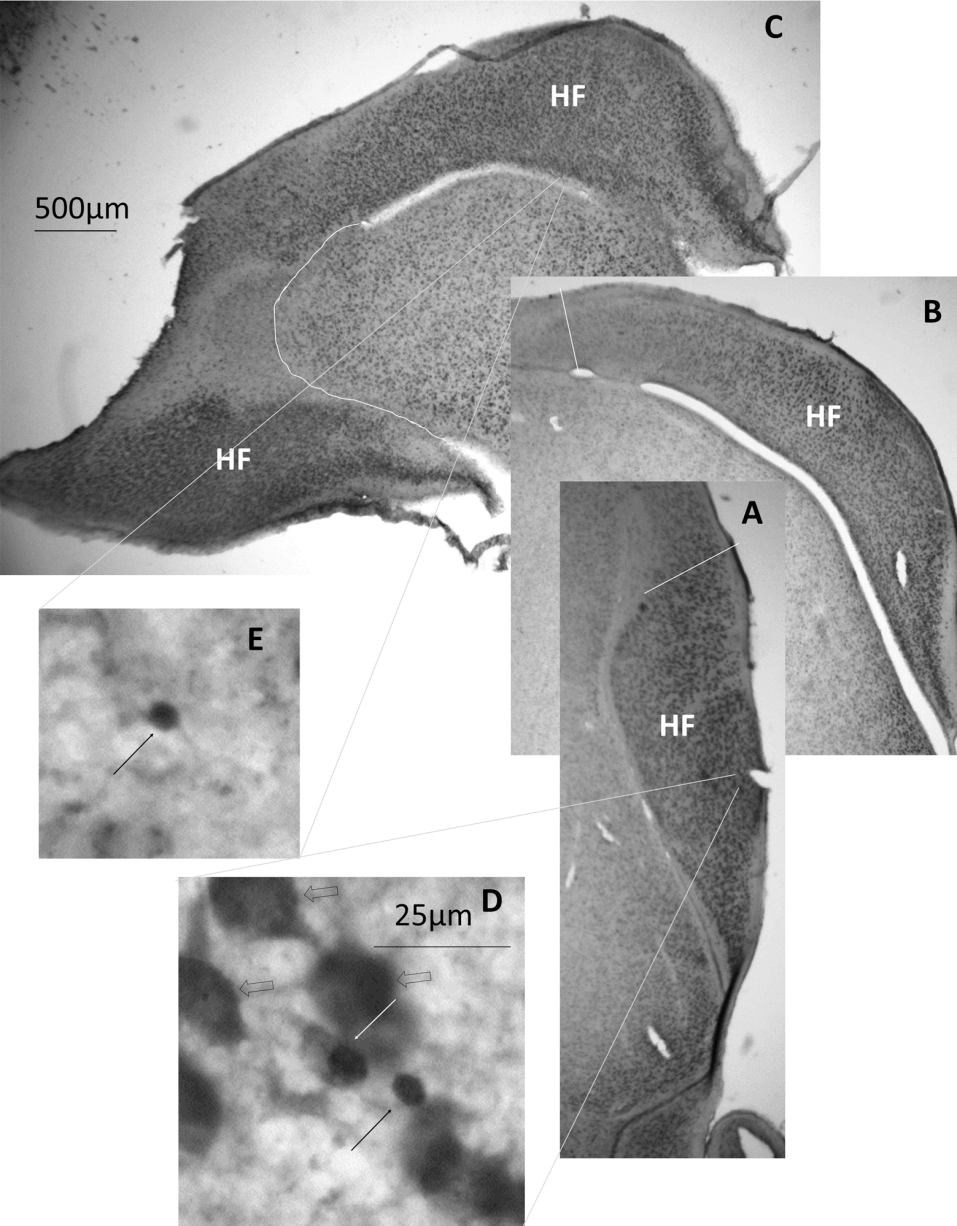
Photomicrographs of the HF at different levels. **A-C** Photomicrographs of a rostral (A), a medium (but still part of the rostral pole; B), and a caudal (C) coronal section through the hippocampal formation (HF). **D.** BrdU^+^/Hu^+^ (white arrow), BrdU^+^/Hu^-^ (black arrow), and BrdU^-^/Hu^+^ (open arrows) cells from the rostral section of the HF. **E.** BrdU^+^ cell in the ventricular wall of the caudal section of the HF. The scale bar in C applies to A and B as well. The scale bar in D also applies to E.

Boundaries were drawn around the telencephalon and HF (Fig 1A–1C) at 2.5x magnification in every 3^rd^ section on the slide (i.e. every 12^th^ 50μm section in the brain) using StereoInvestigator 9.10.1 attached to a Leica DM-LB microscope. Telencephalon and HF volumes were calculated by multiplying the surface areas of the sections by the distance between the sections (50 μm x12 = 600 μm) and adding up these mini-volumes. Because of the irregular shape of the HF and the slight differences in cutting angles, in some brains, the last hippocampal section was quite large, because it was cut through the caudal pole of the telencephalon, which in chickens is covered by the HF (e.g. Fig 1C). This large section was missing from some other brains, because it was not present in the sample of one section every 200 μm. We used the presence or absence of this final section as a factor in the ANOVA to control for the extra variation caused by this inconsistency. Two different researchers outlined the brain areas and counted neurons, one did 8 birds (4 FR and 4 AL) and the other 16 (8 FR and 8 AL). We also used the person counting the cells as an ANOVA factor to control for subtle differences in interpretation of the criteria.

Total hippocampal neuron numbers (Hu^+^; Fig 1D) were estimated stereologically in the outlined sections using the Optical Fractionator (100x magnification (oil immersion); counting grids of 50μm x 50μm, regularly spaced at 450μm intervals; mounted thickness estimated at 15μm). We obtained Gundersen coefficients of error (m = 1) <0.06 for all estimates. Neuronal density was calculated as the total neuron numbers divided by the total estimated volume of the HF.

To estimate the density of new neurons, we counted each BrdU-positive cell in every other outlined HF section (i.e. 1200 μm apart) using the Meander Scan function in StereoInvestigator. We counted three categories of cells: BrdU^+^/Hu^-^ cells in the ventricular zone (Fig 1E), BrdU^+^/Hu^-^ cells in the rest of the HF, and BrdU^+^/Hu^+^ cells in the rest of the HF (Fig 1D). These last ones represent the newly-generated neurons. In order to calculate the cell density, the numbers were divided by the volume represented by the sampled sections. For the cells at the ventricular zone, the numbers were divided by the length of the ventricular zone along which they had been counted.

In order to investigate new neuron density in the rostral and caudal poles of the HF separately, we defined the caudal HF as starting where the HF starts wrapping around the rest of the telencephalon in coronal sections, and therefore where part of the hippocampal tissue can be found in the ventrolateral part of the telencephalon as well at the dorsomedial end of the section [70] (Fig 1C). Because the double-labelling had been performed in different staining batches, with different levels of BrdU staining, we used staining batch as a random factor in any analyses of BrdU-labelled cells. Each staining batch contained equal numbers of AL and FR birds.

### Corticosterone measurements

CORT was assayed in plasma samples using an ELISA kit (Enzo Life Sciences, Exeter, UK) as we have previously described for chickens [71]. A 1:20 dilution of plasma was used in combination with a 2% concentration of steroid displacement reagent.

### Quantitative gene expression analysis

RNA extraction from frozen tissue samples and cDNA synthesis were performed as we have described previously [63], except that adrenal and spleen tissue samples were disrupted using a Polytron homogenizer (Kinematica, Eschbach, Germany). Gene expression was quantified using real-time PCR as described in Dunn *et al*. [63]]. Pro-opiomelanoctin (*POMC*) and the lamin B receptor housekeeping gene cDNAs were amplified with primers we have described previously [63]. The primers used to amplify ADP ribosylation factor like GTPase 10 (*ARL10*) cDNA were those described by Bureau *et al*. [72]]. Steroidogenic acute regulatory protein (*STAR*) cDNA was amplified using forward primer 5’-GGCTTCTTAGCATCGACCTG (positions 956–975 of NM_204686) and reverse primer 5’- CCCTGACCAAAGCACTCAAT (positions 1096–1114); and for interleukin 6 (*IL6*) the forward primer was 5’- GGCTTCGACGAGGAGAAATGCCT (positions 398–420 of NM_204628) and the reverse primer 5’- GCGGCCGAGTCTGGGATGAC (positions 578–597).

### Statistical methods

The General Linear Model and Linear Mixed Model analyses were used in SPSS Version 22. Log transformation of the corrected values was used where appropriate. Several covariates were employed to control for differences due to body mass, experimenter drawing outlines, histological staining batch (used as a random factor), and telencephalon volume (when hippocampal volumes were compared). Negative results are also presented, when appropriate. *Post hoc* analyses, when conducted, used Least Significant Differences. The level of significance was set at α = 0.05. Descriptive statistics are expressed as mean ± SEM. The data can be found in the supplementary materials.

## Results

**Fig 2:**
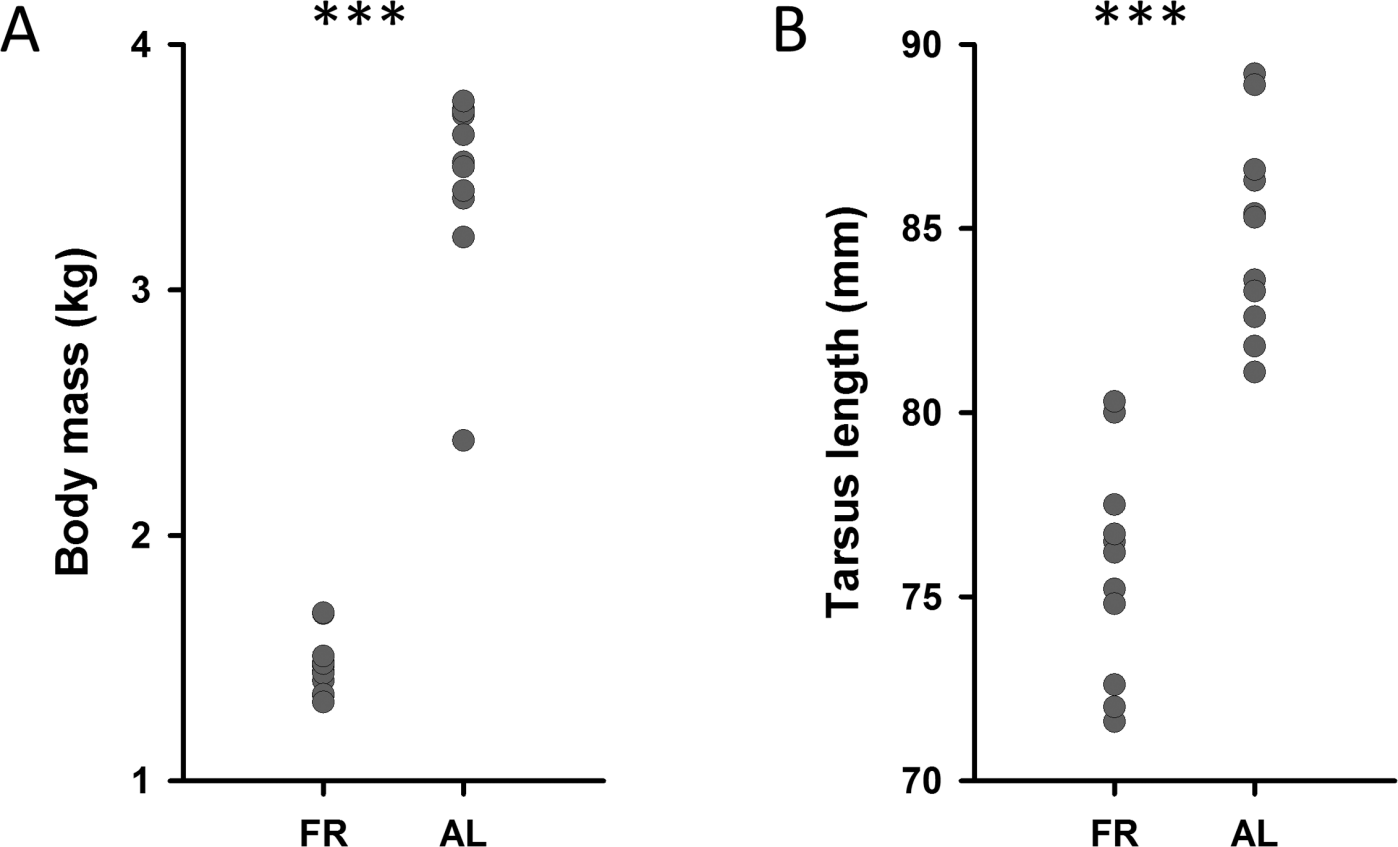
Body size. FR birds had lower body mass (A; 1467±81g) and shorter tarsometatarsal length (B; 75.4±0.8mm) than AL birds (3458±81g and 85.3±0.8mm resp.).*** p < 0.001.

The raw data presented here can be found in the Supplementary Materials (S1 File). FR chickens were smaller and lighter than AL chickens, with shorter tarsometatarsals (F(1, 23) = 67.880, p< 0.001) and lower body mass (F (1,23) = 304. 909, p < 0.001; Fig 2). AL birds had a larger pituitary gland than the FR birds (F(1, 22) = 10.807, p = 0.003). This difference, however, is completely accounted for by the difference in body mass between the two groups, as controlling for body mass removed this effect (F(1,21) = 0.014, p = 0.91; body mass as covariate: F(1,21) = 0.417, p = 0.525). The adrenal gland mass was also larger in the AL than in the FR birds (F(1,21) = 65.150, p<0.001), but again, this was accounted for by the differences in body mass between the two groups (F(1,20) = 1.45, p = 0.243 when including body mass as a covariate).

**Fig 3:**
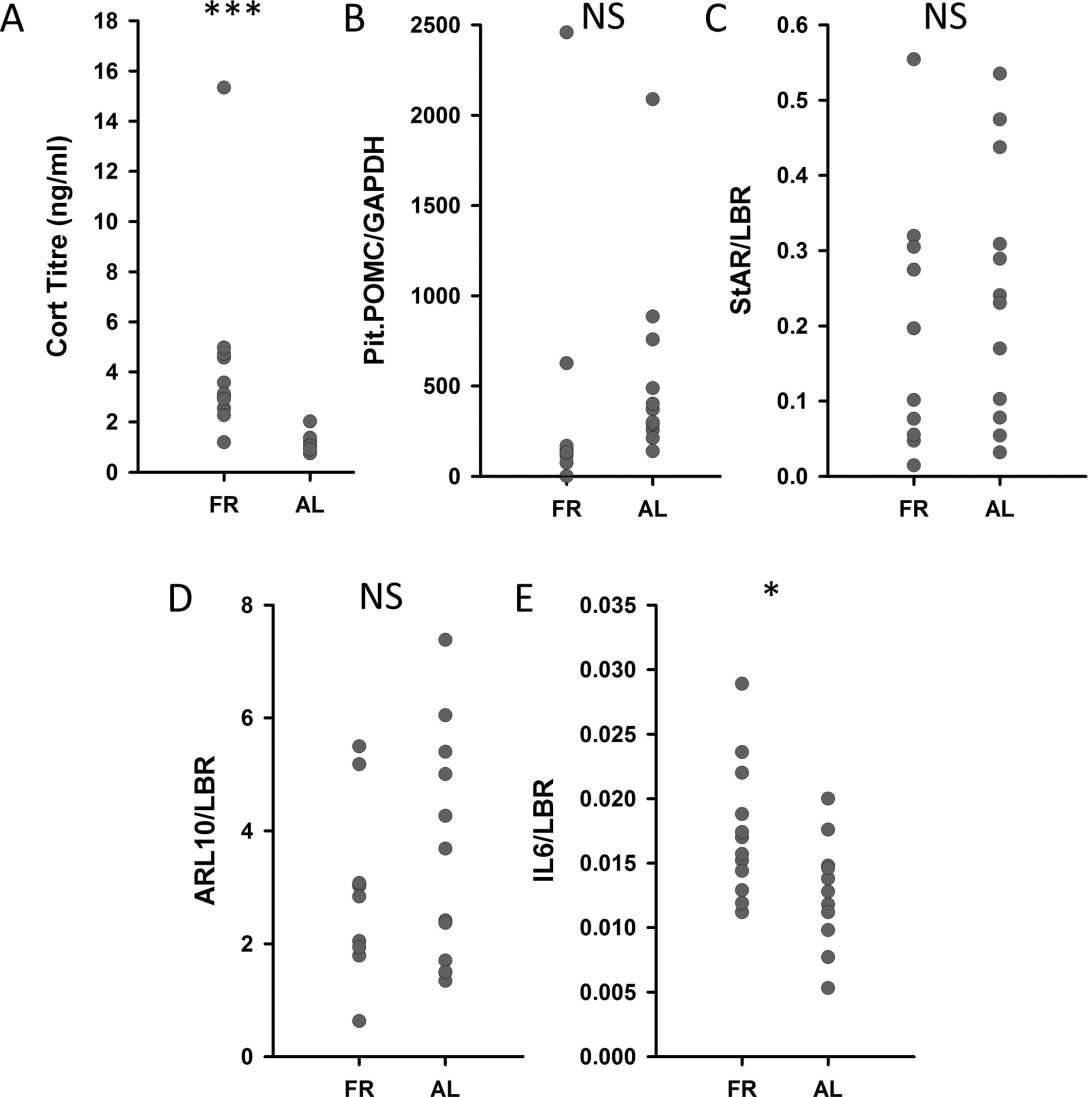
Endocrinological and molecular stress markers. FR birds had higher baseline CORT levels in plasma than AL birds (A). There were no differences in the expression of pro-opiomelanocortin (*POMC*) in the pituitary gland (B), nor of steroidogenic acute regulatory protein (*STAR*) (C) and ADP ribosylation factor like GTPase 10 (*ARL10*) (D) in the adrenal glands. However, Interleukin-6 (*IL6*) expression in the spleen was significantly higher in FR than in AL birds (E). *** p<0.001; * p<0.05.

CORT titres were higher in the FR than in the AL birds (F(1, 22) = 30.40, p < 0.001; Fig 3A). There were no significant differences between the FR and AL groups for expression of pituitary *POMC* (F(1,19) = 0.560, p = 0.464; Fig 3B) or for *STAR* (F(1, 20) = 0.812, p = 0.379; Fig 3C) and *ARL10* (F(1,20) = 0.170, p = 0.685; Fig 3D) in the adrenal gland. *IL6* expression in the spleen was significantly higher in the FR than in the AL birds (F(1,21) = 5.71, p = 0.026; Fig 3E).

**Fig 4:**
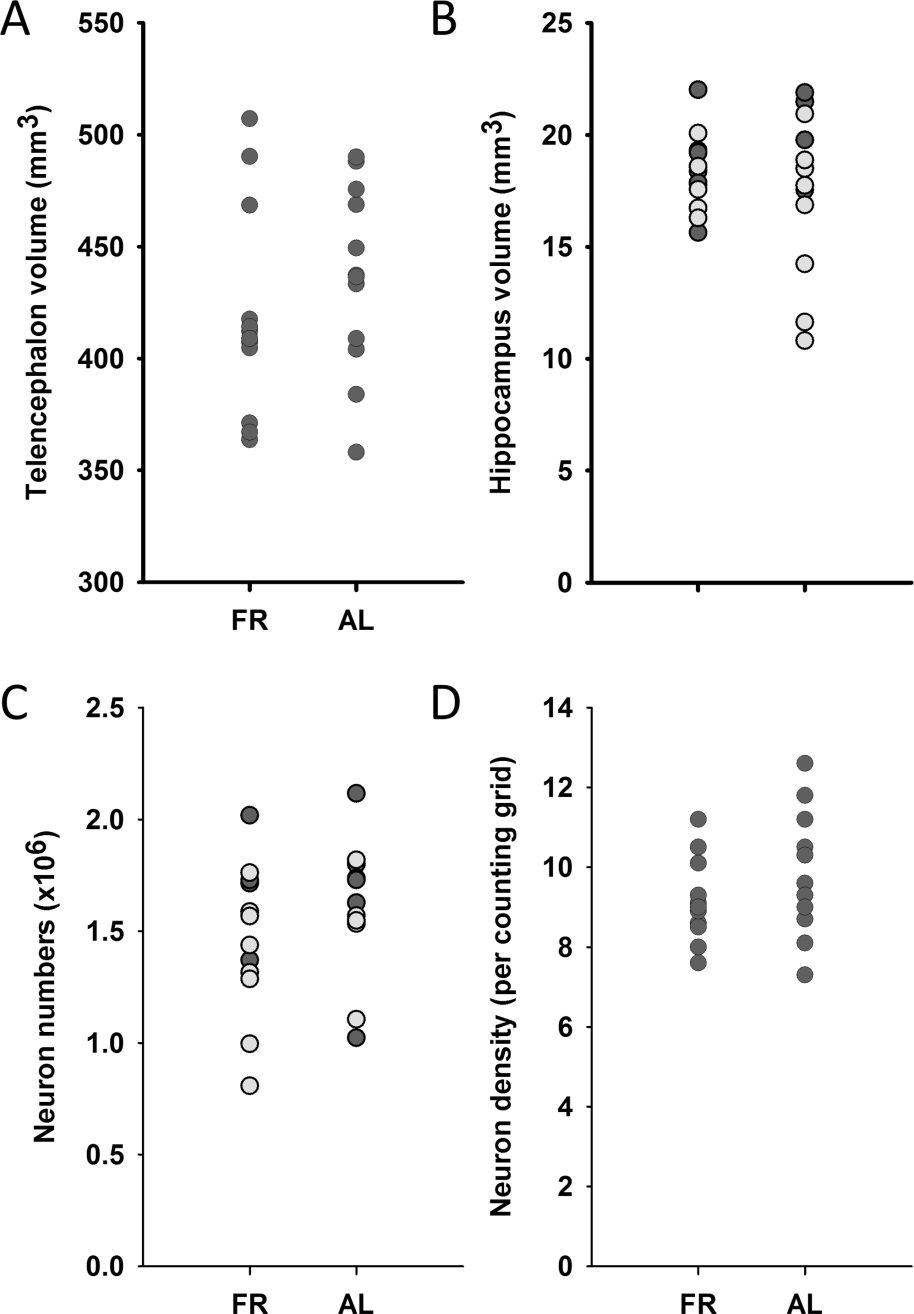
Brain size measures. There were no differences between FR and AL birds in telencephalon volume (A), hippocampal volume (B), total number of hippocampal neurons (C) or density of hippocampal neurons (D). In panel B and C, values from birds for which the caudal-most section of the HF was present are dark, and those for which that section was missing are plotted in a lighter shade.

Chickens in the FR treatment had similar absolute telencephalic volumes (minus the HF) compared with AL chickens (F(1, 21) = 1.16, p = 0.294; Fig 4A), controlling for the experimenter who drew the outlines. Note that this was not controlled for body size, which clearly differed between the two groups. Taking into account which experimenter took the measurement, and whether the caudal pole was present or not, hippocampal volumes also did not differ between the two groups, whether controlling for total telencephalon volume (F(1, 19) = 0. 226, p = 0.640) or not (F(1,20) = 0.145, p = 0.707), although, when controlling for telencephalon size, the lack of caudal pole did make the estimate smaller, as would be expected (F(1,19) = 8.98, p = 0.007; Fig 4B). Controlling for the same potential confounding variables as for the hippocampal volume analysis, there were no differences between the two treatment groups in total hippocampal neuron numbers (F(1,20) = 0.341, p = 0.566; Fig 4C) or in hippocampal neuronal density (F(1,20) = 1.38, p = 0.254; Fig 4D).

**Fig 5:**
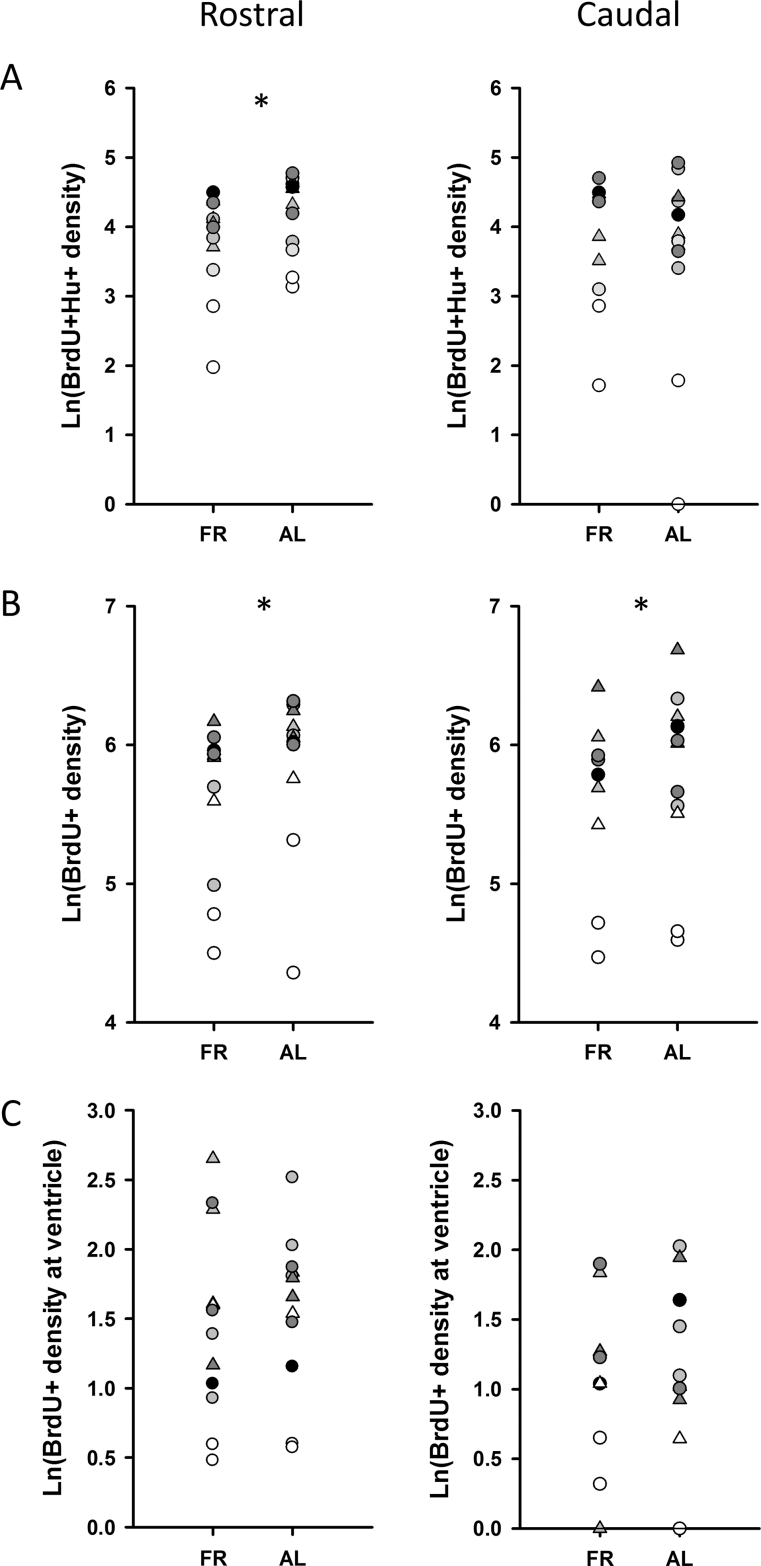
Neurogenesis. (A) There were significantly more BrdU^+^/Hu^+^ new neurons in the rostral HF of AL birds than FR birds. This trend was not significant in the caudal pole of the HF. (B) There were significantly more BrdU^+^/Hu^-^ cells in the AL than in the FR birds, in both the rostral and caudal poles of the HF. (C) There is no effect of treatment on the number of BrdU^+^ cells in the ventricular zone, but there are more ventricular BrdU^+^ cells in the rostral than in the caudal HF, and this effect is the same for both treatments. Different symbols and shades of grey represent brains that were stained in different staining batches. Staining batch was used as a random factor in the Linear Mixed Model. * p<0.05.

There was a significant interaction between feeding treatment and hippocampal pole in the analysis of the density of new hippocampal neurons (F(1,19) = 7.68, p = 0.012). Density of BrdU^+^/Hu^+^ cells was reduced in FR compared to AL birds in the rostral pole of the HF (F(1,16) = 10.94, p = 0.004), but not in the caudal pole (F(1,14) = 0.26, p = 0.62; Fig 5A). New non-neuronal hippocampal cells were also less abundant in FR birds (F(1,12) = 6.09, p = 0.029), and this did not differ between the two poles (interaction: F(1,17) = 0.99, p = 0.34; Fig 5B). There were more BrdU^+^ cells in the lateral ventricle of the rostral pole of the HF than in the caudal pole (F(1,22) = 18.90, p<0.001), but no treatment differences were found in the density of BrdU^+^/Hu^-^ cells (F(1, 15) = 0.19, p = 0.67), in either pole (interaction: F(1,22) = 0.081, p = 0.78; Fig 5C).

## Discussion

Compared to chickens released from commercial food restriction from weeks 7–12 of life, chickens on a commercial food-restricted diet showed elevated CORT levels in blood plasma, increased *IL6* expression, reduced numbers of non-neuronal cells in the entire HF, and reduced neurogenesis in the rostral HF. Feeding condition, however, did not affect telencephalon or hippocampal volume, nor total neuronal density and number in the HF. Along the lateral ventricle, the site of cellular proliferation [9], there was no difference in the density of recently divided cells.

Reduced overall mass and tarsometatarsal length provide evidence that the food restriction treatment was effective: food-restricted birds were both lighter and smaller than their counterparts fed *ad libitum* and were within the range of the growth trajectory set out in the broiler-breeder management manual (see Methods). Despite the fact that somatic growth was delayed compared to the AL birds, brain size (telencephalon volume, hippocampal volume and hippocampal neuron numbers) did not differ between the two groups. This suggests that the birds, despite growing more slowly, were not starved, and brain development continued normally in both groups of birds.

The main finding of our study is that hippocampal neurogenesis (or at least the 1-week survival of newly-generated neurons in the avian HF) is down-regulated in food-restricted adolescent broiler breeder hens, while the population of precursor cells did not differ. This is similar to the results of Cardoso *et al*. [58]], who found that food restriction decreased the number of maturing neurons in the dentate gyrus of adolescent rats, but not the number of dividing cells. It is, however, in contrast to the findings by Lee and colleagues in adult rats [54] and mice [55, 56], which show an increase in neurogenesis across the dentate gyrus when they had their *ad libitum* food supply removed on alternate days. These findings may identify a potentially sensitive developmental stage in both mammals and birds, during which the HF is sensitive to food restriction. Whether food restriction would increase hippocampal neurogenesis in adult chickens remains to be seen. Alternatively, Lee et al.’s rats and mice were able to compensate for the alternating days’ feeding schedule by feeding more on the days when food was available. This option was not available to our animals or to Cardoso et al.’s rats, and could therefore lead to different results. It would be interesting to contrast these two conditions in relation to neurogenesis in chickens.

### Why does food restriction suppress hippocampal neurogenesis?

One possibility is that the reduction in hippocampal neurogenesis due to food restriction is driven by an energetic constraint: the food-restricted animals have a lower energy intake, and therefore they cannot support as many new neurons as the *ad libitum* fed animals, especially while they are still growing and developing. Indeed, Cardoso *et al*. [58]] speculate that their finding that hippocampal neurogenesis in young rats is reduced by food restriction may be due to an increased sensitivity to protein restriction during development. However, even though our animals had restricted food intake, they were still growing at a “normal” rate for chickens (if not for broilers), indicating that they are not in a state of starvation. More importantly, telencephalon size, hippocampal size and total hippocampal neuron numbers did not differ between the two groups, suggesting that brain development was protected, despite differences in body growth. Similar results were also found in the adolescent rats [58].

A related possibility is that certain growth factors involved in the regulation of hippocampal neurogenesis are affected by the amount of food animals have access to. For example, plasma insulin-like growth factor 1 (IGF1) concentrations are decreased by food restriction in broiler chickens, and increased by re-feeding [73]. We did not measure IGF1, but it is likely that in our animals as well, this growth factor would differ between the two treatments. In rats, peripheral IGF1 treatment increases the number of newly-born neurons in the dentate gyrus of the hippocampus [74]. We did not find a difference in the density of dividing cells in the ventricular zone in our study, but IGF-1 might have different effects in birds. Insulin-like growth factor 2 (IGF2) is known to be expressed at times and in brain areas of increased neurogenesis in bird brains [75], so it is possible that IGF-1 might be as well. Similarly, other neurotrophic factors could link food availability to neurogenesis. We do not know whether the effect we observed was specific only to the HF, or whether food restriction also affected neurogenesis in other parts of the avian forebrain. This remains a question for the future.

Another possibility is that the decrease in hippocampal neurogenesis in our birds was a response to chronically increased CORT titres, which can suppress adult hippocampal neurogenesis in rodents [11, 12]. Indeed, despite the fact that the relative size of the pituitary did not differ between the two groups in our study, and neither did the expression of a number of key genes in the HPA axis, FR birds clearly had higher CORT blood titres than AL birds. This is consistent with previous findings in food-restricted broilers [76] and other birds, in which CORT titres increase in response to acute [77, 78] and chronic [79] food restriction. High CORT levels have functional importance in promoting the recovery from metabolic stressors by mobilizing energy stores via the stimulation of proteolysis and gluconeogenesis [80]. It is therefore possible that reduced neurogenesis is a side-effect of the increased CORT titres. Cardoso *et al*. [58] did not measure CORT in their animals. However, in a previous study using the same food restriction treatment, they showed a shift in the circadian CORT peak to right before the predicted feeding time, but not a continued increase in CORT levels throughout the day [81]. Nevertheless, they observed a similar reduction in hippocampal neurogenesis. This may indicate that this reduction is not due solely to an increase in CORT titres.

Indeed, increased CORT levels are not always associated with decreased neurogenesis. Certain types of stress, particularly those positive in nature, can stimulate neurogenesis and other protective processes in rodents [82, 83]. This increase in neurogenesis is also dependent on an increase in CORT levels [30]. In birds as well, a mild elevation of CORT levels improves spatial memory performance and does not affect hippocampal volume or neuron numbers [84, 85]. Increased CORT levels associated with chronic negative stress, however, cause a decrease in hippocampal neurogenesis [19, 26, 27, 30, 86–88]. Lehmann *et al*. [30] reported that CORT release in mice given environmental enrichment after experiencing stressful conditions promoted neurogenesis, thereby restoring previous hippocampal damage and providing stress-resilience. In the same study, however, increased CORT associated with social defeat suppressed adult hippocampal neurogenesis. This suggests that even if increased CORT titres are directly responsible for the reduction in survival of new hippocampal neurons in food-restricted adolescent chickens and rats, this would only happen if this increase in CORT levels happened in a negative context [30]. It is unknown which other signalling pathways would be responsible for the differentiation between positive and negative stressors in the HF, but one possibility is the concept of the inverted U response, in which moderate increases have positive effects, while higher levels have negative ones [89].

The final possibility is therefore that the food restriction and associated feelings of hunger are experienced as a chronic psychological stressor. Our chickens are from a breed that has been strongly selected for efficient conversion of food to muscle mass and growth rate. When food restricted, they show strong physiological evidence of hunger [63], and food-restricted broiler breeders have been shown to be very motivated to forage and eat [62]. The frustration of not being able to feed when so highly motivated to do so may act as a strong negative psychological stressor. In rats as well [58], it is possible that hunger is experienced as a more severe psychological stressor, especially during adolescence, when the energy needs are more urgent. The only other finding in our data that supports this interpretation is that *IL6* gene expression was upregulated in the spleen of food-restricted birds. IL6 biosynthesis is commonly upregulated in human patients suffering from major depressive disorder [90], and increased circulating IL6 protein was reported in chronically distressed domestic chicks [91]. *IL6* gene expression was increased in lymphocytes by externally-administered CORT in laying hens [92], but this increase was temporary, and not detectable after 1 week of CORT administration. This suggests that the increased *IL6* mRNA were observed after chronic food restriction might have been due at least partially to CORT-independent mechanisms.

### Implications for homologies between the avian and mammalian hippocampal formations

As in mammals, we show here that in birds as well, a reduction in the number of newly-generated neurons in the hippocampus accompanies food restriction during adolescence and/or a chronic increase in CORT concentrations in a presumably negative environment. Proliferation of the precursors (here in the ventricular zone) is not changed by these conditions, but survival (here for one week) after proliferation is increased.

Interestingly, this effect was strongest in the rostral HF, and not detectable in the caudal HF. Given that the rostral HF has been hypothesized to be equivalent to the rodent dorsal hippocampal pole, and the caudal HF to the ventral pole [42], and the rodent ventral pole is more sensitive to stressors than the dorsal pole [22], this finding contradicts that hypothesis. However, we should not be too quick to throw out the hypothesis yet. In rodents, the dorsal pole also sometimes responds to chronic stress and/or increased CORT levels [93, 94], and in rare occasions this even happens without a response in the ventral pole [95]. It should also be pointed out that in coronal sections, the caudal pole of the avian HF is parallel to the cutting plane, and therefore represented in many fewer sections, so that our sampling of the rostral pole is much more robust than that of the ventral pole, leading to poorer statistical power. Note also that the difference cannot be explained by differences in activity or spatial experience, because FR birds are more active and constantly exploring the environment, looking for food, while the AL birds spend most of their time lying still. An experience-dependent increase in neurogenesis, as often demonstrated in rodents [83, 96, 97], should therefore have favoured the FR group, and no the AL group.

The similarity in the response of avian and mammalian adult hippocampal neurogenesis to chronic stress indicates that this may be an ancient feature of the structure, inherited from the last common ancestor more than 300 million years ago. More research is needed to better understand the similarities and differences between the avian and mammalian HF.

## Conclusion

In conclusion, the evidence in our study suggests that avian hippocampal formation is similar in its response to at least one type of chronic stressor (or at least to physiologically increased CORT levels) to the mammalian hippocampus. This is likely to represent a conserved feature of hippocampal structure and function.

## Supporting information

S1 File Analysed data. This file contains all the data reported in the results section and presented in the figures.
(XLSX)
